# Silencing of *CHD5* Gene by Promoter Methylation in Leukemia

**DOI:** 10.1371/journal.pone.0085172

**Published:** 2014-01-13

**Authors:** Rui Zhao, Fanyi Meng, Nisha Wang, Wenli Ma, Qitao Yan

**Affiliations:** 1 Institute of Molecular Biology, Southern Medical University, Guangzhou, PR China,; 2 Department of Hematology, Nanfang Hospital, Southern Medical University, Guangzhou, PR China; University of Navarra, Spain

## Abstract

Chromodomain helicase DNA binding protein 5 (CHD5) was previously proposed to function as a potent tumor suppressor by acting as a master regulator of a tumor-suppressive network. *CHD5* is down-regulated in several cancers, including leukemia and is responsible for tumor generation and progression. However, the mechanism of *CHD5* down-regulation in leukemia is largely unknown. In this study, quantitative reverse-transcriptase polymerase chain reaction and western blotting analyses revealed that *CHD5* was down-regulated in human leukemia cell lines and samples. Luciferase reporter assays showed that most of the baseline regulatory activity was localized from 500 to 200 bp upstream of the transcription start site. Bisulfite DNA sequencing of the identified regulatory element revealed that the *CHD5* promoter was hypermethylated in human leukemia cells and samples. Thus, *CHD5* expression was inversely correlated with promoter DNA methylation in these samples. Treatment with DNA methyltransferase inhibitor 5-aza-2′-deoxycytidine (DAC) activates *CHD5* expression in human leukemia cell lines. *In vitro* luciferase reporter assays demonstrated that methylation of the CHD5 promoter repressed its promoter activity. Furthermore, a chromatin immunoprecipitation assay combined with qualitative PCR identified activating protein 2 (AP2) as a potential transcription factor involved in *CHD5* expression and indicated that treatment with DAC increases the recruitment of AP2 to the *CHD5* promoter. *In vitro* transcription-factor activity studies showed that AP2 over-expression was able to activate *CHD5* promoter activity. Our findings indicate that repression of *CHD5* gene expression in human leukemia is mediated in part by DNA methylation of its promoter.

## Introduction

Previous studies report that tumors and cell lines commonly harbor deletions in genes that encode proteins protecting against malignancy. For example, a deletion in human chromosome 1p36, first reported for a neuroblastoma in 1977 [1], has been associated with cancer. This discovery precipitated a rush of studies demonstrating that 1p36 is deleted in a broad range of human cancers, including those of neural, epithelial and hematopoietic origin. Neural-related malignancies associated with 1p36 deletion include neuroblastoma [1], [2], [3], meningioma [4], melanoma [5], pheochromocytoma [6] and oligodendroglioma [7]. 1p36 deletions are also associated with hematopoietic malignancies, including acute myelogenous leukemia (AML) [8], chronic myelogenous leukemia (CML) [9] and non-Hodgkin’s lymphoma [10], as well as with epithelial malignancies, including those of the thyroid [11], colon [12], cervix [13] and breast [14]. These data suggest that one or more tumor suppressor genes located in 1p36 are lost or inactivated in a variety of human cancers.

Based on sequence similarity with members of the chromodomain superfamily of proteins, the human chromodomain helicase DNA binding protein 5 (CHD5) is believed to function as a chromatin remodeling protein [15]. CHD5 is one of nine members of the family, which are characterized the unique combination of chromatin organizing modulator, helicase and DNA-binding domains. In addition, *CHD5*, *CHD3* and *CHD4* also have plant homeodomain (PHD) motifs [16].

CHD3 and CHD4 are components of the nucleosome remodeling complex; an assembly of proteins that remodels chromatin by sliding nucleosomes out of the way, thereby providing polymerase with the access needed to activate gene expression. Other than this homology between CHD5 and previously described chromatin remodeling proteins, little functional data existed for this protein. Further supporting the role of *CHD5* in cancer, the mouse Chd5 has been identified as a tumor suppressor.

*CHD5* expression has been shown to be epigenetically silenced by promoter hypermethylation in a variety of human cancers, including neuroblastomas [17], colorectal cancer [18], breast cancer [19], cervix cancer [19], hepatocarcinoma [19], gastric cancer [20] and lung cancer [21]. In addition, *CHD5* mutations have been implicated in head and neck squamous cell carcinoma [22], human prostate cancer [23], ovarian cancer [24], ovarian clear cell carcinoma [25], cutaneous melanoma [26], hepatocellular carcinoma [27], neuroblastomas [28], metastatic prostate tumors [29], breast cancer and colorectal cancers [30]. Furthermore, KDM4A lysine (K)-specific demethylase 4A (KDM4A) has been shown to inactivate *CHD5* expression through transcriptional repression [31].

In addition to regulating processes that are fundamental for cancer prevention, *CHD5* expression is also a favorable predictor of survival following anticancer therapy [17], [32], [33], [34]. Several results indicate that the chromatin remodeling function of *CHD5* might be important for preventing cancer. Loss of Chd5 enhances proliferation; whereas Chd5 gain compromises proliferation [35]. Restoration of *CHD5* expression inhibited proliferation and tumor growth in neuroblastoma [36], breast cancer [37] and lung cancer [21] cells. *CHD5* facilitates expression of a tumor-suppressive network that includes p16 and p19, encoded by the *cyclin-dependent kinase inhibitor 2A* (*Cdkn2a*) locus [35]. The ability of *CHD5* to bind unmodified histone 3 (H3) is essential for tumor suppression [38]. Mutation of the *CHD5* PHD domain abrogated CHD5–H3 interaction and consequently compromised proliferation inhibition, inducing differentiation and suppressing tumorigenesis [38]. CHD5 binds and regulates an extensive number of cancer-associated loci, including tumorigenic *Cdkn2a*, and other genes encoding proteins implicated in chromatin dynamics and cancer-associated pathways. These data taken together strongly suggest that *CHD5* modulates the expression of multiple genes regulating pathways involved in tumorigenic processes [38]. Excessive activation of these tumor-suppressive pathways causes apoptosis, cellular senescence, and neonatal death, all of which are dependent on p16, p19 and p53 [35].

Deletions of regions including Chd5 in a mouse model of chromosomally unstable lymphoma showed that CHD5 is one of only three genes mapping to the minimally common region of deletion that they discovered in human T-cell acute lymphoblastic leukemia/lymphoma [39]. However, deletions of the human 1p36 region identified for several hematopoietic malignancies, including acute AML [8], CML [9] and non-Hodgkin’s lymphoma [10], did not include *CHD5*. Although this does not support CHD5 deficiency as a causal event in these cancers, the finding that mice heterozygous for the 4.3-Mb interval encompassing *Chd5* are prone to spontaneous lymphoma [35] and that *CHD5* is deleted in lymphoid cancers in mice provide evidence that CHD5 deficiency plays a key role in these hematopoietic malignancies [39].

Given the pivotal role of *CHD5* in leukemia, we hypothesize that *CHD5* is inactivated in leukemia and sought to elucidate the mechanism of inactivation. Here we demonstrate the following: *CHD5* was down-regulated in human leukemia cell lines and samples, the important regulatory region of the *CHD5* gene is localized 500–200 bp upstream of the transcription start site (TSS), hypermethylation was observed in these important regulatory elements, and the hypermethylation of the *CHD5* promoter repressed transcriptional activity. Furthermore, the activating protein 2 (AP2) binding site was identified as a region strongly regulating *CHD5* expression. Our findings indicate that repression of *CHD5* gene expression in human leukemia is mediated in part by hypermethylation of the identified *CHD5* regulatory element.

## Materials and Methods

### Human tissues and cells

Human leukemia cell lines K-562, HL-60, KG-1a and Jurkat were purchased from American Type Culture Collection (Manassas, VA) and were maintained in RPMI 1640 medium (Gibco, Grand Island, NY, USA) supplemented with 10% fetal bovine serum (Gibco). Bone marrow obtained from 12 healthy donors, 50 acute lymphoblastic leukemia (ALL) patients, 50 AML patients and 50 chronic CML patients (Table S1) was donated by Southern Hospital,Guamgzhou, China. These leukemic samples were selected randomly from primary patients. The Southern Hospital Institutional Review Board approved this study, and written informed consent was obtained before sample collection. Mononuclear cells (MCs) were separated from bone marrow using Ficoll-Paque PLUS (GE Healthcare, Piscataway, NJ, USA) according to the manufacturer’s instructions, and used for analysis of CHD5 expression and methylation status.

### Quantitative reverse transcriptase-polymerase chain reaction (qRT-PCR)

Total RNA was prepared using Trizol reagent (Invitrogen, Carlsbad, CA, USA) according to the manufacturer’s instructions. RT-PCR was performed on 0.5 mg total RNA using a PrimeScript RT Reagent Kit with gDNA Eraser (Takara, Japan). qPCR was performed using an ABI 7500 Real-Time PCR system and a SYBR Premix Ex TaqTM II kit (Takara). Gene expression of CHD5 was normalized to β-actin and all samples were analyzed in triplicate. Primers for qRT-PCR are shown in Table 1.

**Table 1:** Primers for qRT-PCR.

Target	Sequence
CHD5 mRNA (NM_015557)	qCHD5-F:5′-TCCGCAAGCAGGTCAACTACA-3′
	qCHD5-R:5′-CTCATCCTCAGAGCCAATGGAA-3′
β-actin mRNA (NM_001101)	qβ-actin-F:5′-TGGCACCCAGCACAATGAA-3′
	qβ-actin-R:5′-CTAAGTCATAGTCCGCCTAGAAGCA-3′

### Western blot analysis

Cells were lysed in Laemmli buffer and protein concentrations were determined using Bio-Rad protein assay kits (Bio-Rad**,** Hercules**,** USA) and 50−100 µg protein was analyzed by using standard procedures, with antibodies specific for CHD5 (1:1000, rabbit monoclonal; Abcam, Cambridge,USA) and β-actin (AC-15, mouse monoclonal, 1:1000; Abcam). Immunoreactive bands were visualized by chemiluminescent detection (ECL detection kit; PEL**,**Arkansas, USA.). β-Actin was included as a loading control. All extracts were prepared in duplicate, and at least three independent experiments were conducted.

### Bisulfite DNA sequencing (BGS)

Human promoter of CHD5 was analyzed using Methyl Primers Express software (Applied Biosystems, Foster City, CA) and MethPrimer to identify CpG islands, and corresponding primers were used for BGS. Genomic DNA was isolated using the genomic DNA isolation kit (Qiagen,Valencia, CA). DNA (1 µg) was converted with EpiTect Bisulfite Kit (Qiagen). BGS primers [5′-GTTGTTTTGAAGATTTTGTTTT -3′ and 5′-CTAATTACTATAACAACCCCATCCC-3'] were used to amplify the CpG island located at 560–240 bp upstream of the TSS. The PCR products were purified using agarose gel electrophoresis and the Qiagen purification kit (Qiagen), and were cloned into the PMD18-T Vector (Takara) for sequencing. A minimum of eight colonies were selected for sequencing. Methylation data from BGS were analyzed using BiQ Analyzer software to generate lollipop diagrams (which list the percentage of methylation at each CpG) and to calculate the efficiency of bisulfite conversion. Analysis of non-CpG cytosines indicated the efficiency of bisulfite conversion was ∼99%.

### Drug treatments

For dose–response experiments, K-562 and Jurkat cells were treated with DNA methyltransferase inhibitor 5-aza-2-deoxycytidine (DAC; Sigma, St Louis, MO, USA) at 0, 0.5, 2.5, 5, 10, and 20 µmol/L for 4 days, and HL-60 and KG-1a cells at 0, 0.1, 0.5, 1, 2.5, and 5 µmol/L. The medium was exchanged every other day with the same concentration of DAC. All experiments were performed in duplicate and repeated twice.

### DNA-based bioinformatics

The location and putative domains of the CHD5 prompter were predicted using the UCSC Genome Browser (http://genome.ucsc.edu/cgi-bin/hgGateway). The promoter was analyzed for transcription factor binding sites (TBSs) using WWW Promoter Scan [40] and TFSEARCH software [41].

### Transient transfection and luciferase assay

The *CHD5* promoter was obtained by whole gene synthesis using the 2000 bp sequence upstream of the TSS. The synthesized promoter was cloned into the pGL4.10 (Promega, Madison, WI, USA), a promoterless luciferase expression vector, to produce the pGL4.10-CHD5-2000 to −1 recombinant vector. A set of reporter constructs containing truncated *CHD5* promoter sequences generated by progressively deleting 200 or 100 bp from the 5' or 3' end were PCR amplified from pGL4.10-CHD5-P-WT and self-ligated. Constructs pGL4.10-CHD5-2000 to −370 and pGL4.10-CHD5-356 to −1, in which the AP2 binding site (located at –369 to –357 bp from the TSS) is deleted, were PCR amplified from pGL4.10-CHD5-2000 to −1. A 321 bp region located at –560 to –240 was PCR amplified from pGL4.10-CHD5-2000 to −1 and the fragments were methylated *in vitro* with *Sss*I, *Hpa*II and *Hha*I methylases (New England Biolabs), or no enzyme (mock), according to the manufacturer’s instructions. *Sss*I methylates all 5′-CpG-3′ sites, *Hpa*II methylates only the CpG within the sequence 5′-CCGG-3′, and *Hha*I methylates only the CpG within the sequence 5′-GCGC-3′. Methylation efficiency was confirmed using *Hha*I, *Hpa*II, and *Mcr*BC digestion (NEB). Methylated or mock-methylated fragments were ligated back into pGL4.10 vector to produce reporter constructs (pGL4.1-CHD5-P-CR for mock-methylated fragment). Primers used to generate these reporter constructs are listed in Table 2.

**Table 2:** Primers for construction of reporter constructs.

Region of the deleted CHD5 promoter (upstream of TST)	Primer	Sequence
−1800bp to −1bp	CHD5-PD1-F	ATGAGCATCTCGGCTCTAATTTGTTG
	CHD5-PD1-R	AGATCTTGATATCCTCGAGG
−1600bp to −1bp	CHD5-PD2-F	TTGGGGGGCTGGTAACTGAG
	CHD5-PD2-R	AGATCTTGATATCCTCGAGG
−1400bp to −1bp	CHD5-PD3-F	GCCTGCTTCCCTGAAGAGTGTT
	CHD5-PD3-R	AGATCTTGATATCCTCGAGG
−1200bp to −1bp	CHD5-PD4-F	GGCAATAAAATTGAACGGGA
	CHD5-PD4-R	AGATCTTGATATCCTCGAGG
−1000bp to −1bp	CHD5-PD5-F	AGGCGCTCATTTAAAATTCC
	CHD5-PD5-R	AGATCTTGATATCCTCGAGG
−800bp to −1bp	CHD5-PD6-F	GGGCTTTTTGGAAGGGGGCA
	CHD5-PD6-R	AGATCTTGATATCCTCGAGG
−600bp to −1bp	CHD5-PD7-F	GTCCCGGCGCCTGTGAACCG
	CHD5-PD7-R	AGATCTTGATATCCTCGAGG
−400bp to −1bp	CHD5-PD8-F	TCGGGTCCGCGGGCGCGCGG
	CHD5-PD8-R	AGATCTTGATATCCTCGAGG
−200bp to −1bp	CHD5-PD9-F	CCGTGCCGCCGTGCCTCTGG
	CHD5-PD9-R	AGATCTTGATATCCTCGAGG
−2000bp to −200bp	CHD5-PD10-F	AAGCTTGGCAATCCGGTACTGTTGGTAAAGC
	CHD5-PD10-R	CGGGGGGGCGGCACACATGC
−2000bp to −400bp	CHD5-PD11-F	AAGCTTGGCAATCCGGTACTGTTGGTAAAGC
	CHD5-PD11-R	CGGCCGAGCGGGCAGTCGGG
−2000bp to −600bp	CHD5-PD12-F	AAGCTTGGCAATCCGGTACTGTTGGTAAAGC
	CHD5-PD12-R	CCACCCCGCTCCCGGCTCCG
−2000bp to −800bp	CHD5-PD13-F	AAGCTTGGCAATCCGGTACTG
	CHD5-PD13-R	CTCCAGGCTGGGTGCAGCTG
−2000bp to −1000bp	CHD5-PD14-F	AAGCTTGGCAATCCGGTACTGTTGGTAAA
	CHD5-PD14-R	CCAGTCTCCGCGGCGGCAAG
−2000bp to −1200bp	CHD5-PD15-F	AAGCTTGGCAATCCGGTACT
	CHD5-PD15-R	AGCAAACCCCAAACAAAAGA
−2000bp to −1400bp	CHD5-PD16-F	AAGCTTGGCAATCCGGTACT
	CHD5-PD16-R	CAGGAAACCAAAGGGCCTCC
−2000bp to −1600bp	CHD5-PD17-F	AAGCTTGGCAATCCGGTACT
	CHD5-PD17-R	CACCTCTCTAATCCCTTTCA
−2000bp to −1800bp	CHD5-PD18-F	AAGCTTGGCAATCCGGTACT
	CHD5-PD18-R	CAGTCATAATCCTCTCCCCA
−2000bp to −700bp and −600bp to −1bp	CHD5-PD19-F	GTCCCGGCGCCTGTGAACCG
	CHD5-PD19-R	AGACTGTGGGTCTGATTTAC
−2000bp to −700bp and −500bp to −1bp	CHD5-PD20-F	CGAGCGGCGGCGGCTGGCTC
	CHD5-PD20-R	AGACTGTGGGTCTGATTTAC
−2000bp to −700bp and −400bp to −1bp	CHD5-PD21-F	TCGGGTCCGCGGGCGCGCGG
	CHD5-PD21-R	AGACTGTGGGTCTGATTTAC
−2000bp to −700bp and −300bp to −1bp	CHD5-PD22-F	TTCTCTGCTGTTAACTAGTC
	CHD5-PD22-R	AGACTGTGGGTCTGATTTAC
−2000bp to −700bp and −200bp to −1bp	CHD5-PD23-F	CCGTGCCGCCGTGCCTCTGG
	CHD5-PD23-R	AGACTGTGGGTCTGATTTAC
−2000bp to −700bp and −100bp to−1bp	CHD5-PD24-F	GCAGGCTAAGGCGGCCGAGA
	CHD5-PD24-R	AGACTGTGGGTCTGATTTAC
−2000bp to −100bp	CHD5-PD25-F	AAGCTTGGCAATCCGGTACT
	CHD5-PD25-R	TCCCCGCAAAGCCCGGGCGC
−2000bp to −200bp	CHD5-PD26-F	AAGCTTGGCAATCCGGTACTGTTGGTAAAGC
	CHD5-PD26-R	CGGGGGGGCGGCACACATGC
−2000bp to −300bp	CHD5-PD27-F	AAGCTTGGCAATCCGGTACT
	CHD5-PD27-R	CAAGTTGGGGGACTAGTCCT
−2000bp to −400bp	CHD5-PD28-F	AAGCTTGGCAATCCGGTACTGTTGGTAAAGC
	CHD5-PD28-R	CGGCCGAGCGGGCAGTCGGG
−2000bp to −500bp	CHD5-PD29-F	AAGCTTGGCAATCCGGTACTGTTGGTAAA
	CHD5-PD29-R	CCTGCGAAGGCGCTGGGCCC
−2000bp to −600bp	CHD5-PD30-F	AAGCTTGGCAATCCGGTACTGTTGGTAAAGC
	CHD5-PD30-R	CCACCCCGCTCCCGGCTCCG
2000bp to −370bp and −356bp to −1bp	CHD5-P-AP-2-D-F	GGGCGCCCCCCGGTCCCCACC
	CHD5-P-AP-2-D-R	CGTCGAGGCGTCCGCGCG
–560bp to –240bp	CHD5-P-CR-F	GCTGCCCTGAAGACCCTG
	CHD5-P-CR-R	CTGGTTGCTGTGGCAACC

Cells were cultured in 24-well plates. Twenty-four hours after seeding, medium was changed and cells were co-transfected with pGL4.10 vector or one of the recombinant pGL4.10 vectors and pGL4.74 (Promega), or co-transfected with pGL4.74 vector, expression vector for human AP-2 (Addgene Plasmid 12100) [42] and pGL4.1-CHD5-P-CR or pGL4.10. The pGL4.10 vector contains luc gene, and the pGL4.74 contains Renilla gene. After 24 hours, cells were harvested, and luciferase activities were measured using the Dual-Luciferase Reporter Assay System (Promega) according to the manufacturer’s instructions. The promoterless pGL4.10 vector (pGL4.10-Basic) was used as a negative control. The pGL4.10-CMV vector, which includes the CMV promoter, was used as positive control for reporter construct experiments. The relative Luc luciferase activity of full length and truncated *CHD5* promoters was calculated by normalizing to Renilla luciferase activity and compared to normalized Luc luciferase activity from the pGL4.10-Basic vector. The experiment was repeated twice to confirm the reproducibility of results.

### Chromatin immunoprecipitation and quantitative PCR

Chromatin immunoprecipitation (ChIP) analysis was performed using the Pierce® Agarose ChIP Kit (Pierce, Waltham, USA). Chromatin was precipitated with an AP2α antibody (#3208, Cell Signaling Technology, Danvers, MA). The abundance of specific DNA sequences in the immunoprecipitates was determined by qPCR. ChIP-qPCR primers ChIP-CHD5-P-F (5'-GCGGGGGCGGTGTTTCCT-3') and ChIP-CHD5-P-R (5'-CAGGGGGAGCCGTCGAGG-3') were used for the region containing the AP2 binding site (–369 to –357). The –468 to –360 region was amplified to determine by qPCR. Samples were run in triplicate, and data were normalized to 10% input DNA amplifications, after subtraction of signals obtained from antibody isotype control. ChIP was repeated twice to confirm the reproducibility of results.

### Statistical analysis

Comparisons between two groups were performed by paired sample *t* tests and between three or more experimental groups by one-way analysis of variance. The correlation between methylation and *CHD5* expression was calculated by Spearman’s correlation. All data are presented as mean ± standard deviation (SD). P<0.001 was considered statistically significant unless otherwise indicated.

## Results

### Expression of CHD5 gene in leukemia cell lines and samples

**Figure 1:**
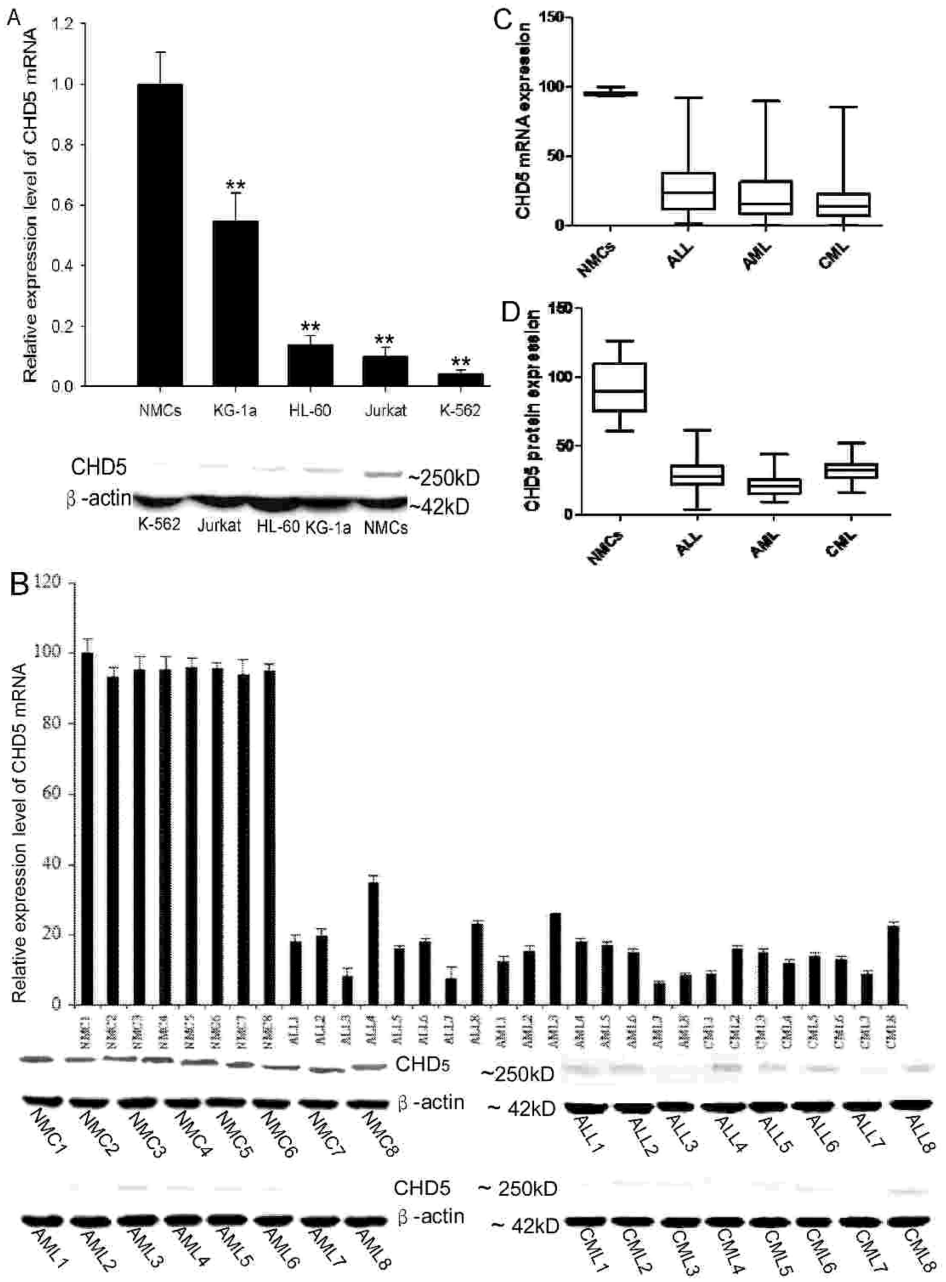
*CHD5* expression is down-regulated in leukemia cell lines and samples. *CHD5* expression was determined for K-562, KG-1a, HL-60 and Jurkat cell lines by qRT-PCR (top) and western blotting (bottom) (A). Representative results of *CHD5* mRNA (top) and protein (bottom) expression from ALL, AML and CML patients are presented (B). The distribution of *CHD5* mRNA (C) and protein level (D) in ALL, AML and CML samples and NMCs (included for comparison) were calculated. For all experiments, β-actin was detected as an internal control. All data are presented as mean ± SD. P<0.001 was considered statistically significant (**).

To determine whether *CHD5* expression was down-regulated in leukemia, we initially examined expression level of *CHD5* mRNA and protein in leukemia cell lines. K-562, HL-60 and Jurkat cells had markedly lower *CHD5* expression relative to normal mononuclear cells (NMCs). However, *CHD5* expression in KG-1a cells was only slightly lower (Figure 1A; P<0.01). Additionally, MCs obtained from bone marrow from leukemia patients were analyzed for *CHD5* expression. In NMCs, the median expression of *CHD5* mRNA was 95.58 (range, 93.333–100). The median expression in ALL, AML and CML was 18.20 (range, 7.666–35), 14.75 (range, 6–25.66) and 13.79 (range, 9–22.333), respectively (Figure 1B). Furthermore, ALL, AML and CML samples had much lower CHD5 protein expression compared to the expression in NMCs (Figure 1B). Statistical analysis revealed that distributions of *CHD5* mRNA and protein expression were significantly lower in ALL, AML and CML than in NMCs (Figure 1 CD; P≤0.001). These data demonstrated that *CHD5* gene expression was down-regulated in leukemia cells.

### Mapping important regulatory elements within the *CHD5* promoter

According to the National Cancer Institute (NCI), the frequency of 1p36 deletion in leukemia samples is less than 5%, and some of these 1p36 deletions do not include the *CHD5* gene. In leukemia cases where the normal *CHD5* gene is intact, the reduction in *CHD5* expression must be due to another mechanism than the absence of the gene sequence. To clarify this issue, we examined transcriptional regulation of *CHD5* expression.

**Figure 2:**
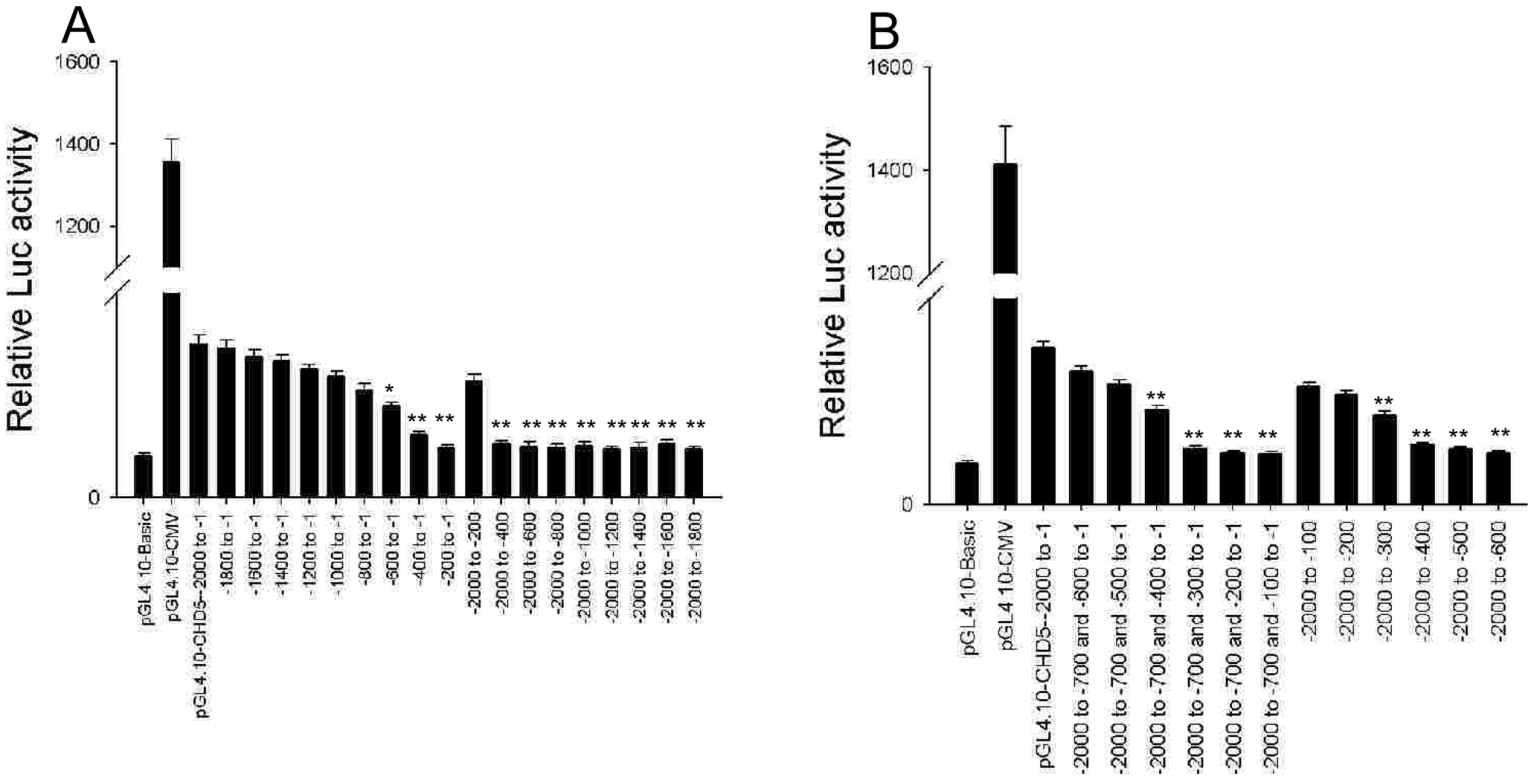
Mapping regulatory elements within the *CHD5* promoter. The reporter constructs containing the full length (–2000 to –1) *CHD5* promoter or partial promoter sequences progressively truncated by 200 bp (A) or 100 bp (B) at the 5' or 3' end were measured for luciferase activity in K-562 cells. The promoterless pGL4.10-Basic vector was used as a negative control. The pGL4.10-CMV vector, which uses the CMV promoter, was used as a positive reporter construct. The relative Luc activity of full length and truncated *CHD5* promoters were normalized to Renilla luciferase activity and compared to normalized Luc luciferase activity from pGL4.10-Basic. All data are presented as mean ± SD. P<0.001 was considered statistically significant (**).

Analysis of the human *CHD5* gene sequence using UCSC Genome Browser software indicated that the putative *CHD5* promoter is located 2000 bp upstream of the TSS and includes a CpG island (Figure S1). To map the regulatory regions important for *CHD5* gene transcription, we cloned full length and truncated *CHD5* promoter sequences into luciferase reporter constructs. Expression from the full length *CHD5* promoter construct was high in K-562 cells compared to expression from the promotorless pGL4.10 vector (Figure 2 A; P<0.01). *CHD5* promoters with deletions in the –600 to –200 region exhibited significant less transcriptional activity than the full length promoter (Figure 2 A; P<0.01). Furthermore, promoter sequences generated by sequential 100 bp truncations of the 5' end starting at –700 bp or of the 3' end starting at the TSS exhibited significantly reduced transcriptional activity when the –500 to –200 region was deleted (Figure 2 B; P<0.01). These data suggest that an important regulatory element is located in the –500 to –200 region upstream of the TSS. Furthermore, this important regulatory element completely overlapped with the putative core *CHD5* promoter located –472 to –222 upstream of the TSS, as predicted by WWW Promoter Scan software [40].

### Methylation status of the *CHD5* promoter in leukemia cell lines

**Figure 3:**
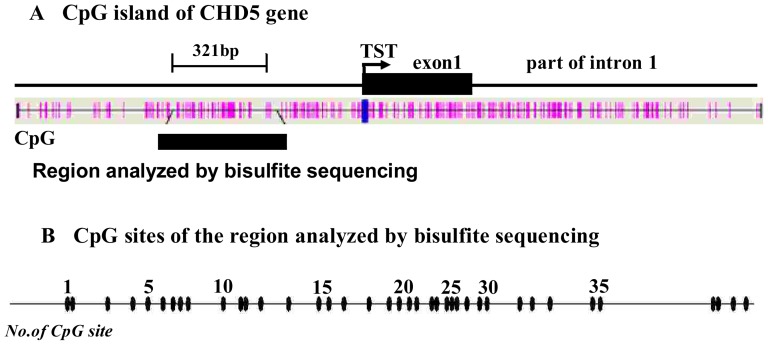
The location of a CpG island (A) and 39 CpG sites analyzed by BGS (B) in the *CHD5* promoter. CpG sites are indicated with pink vertical bars. The positions analyzed by BGS are indicated with black vertical bars. The TSS is indicated with an arrow.

**Figure 4:**
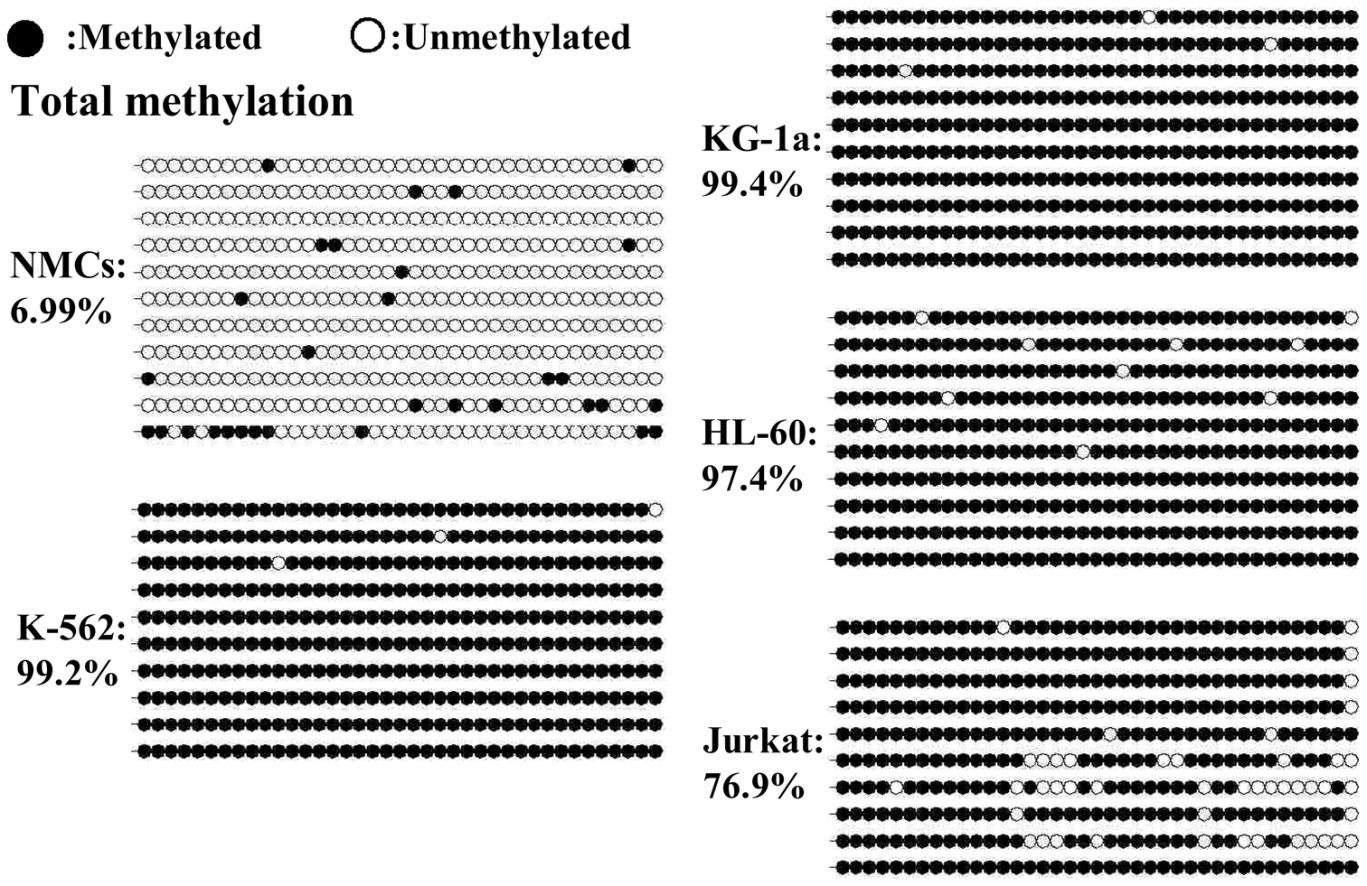
The methylation status of the –560 to –240 region of the *CHD5* promoter. The CpG island, located at –560 to –240, was analyzed by BGS. Methylation data from BGS were analyzed by BiQ Analyzer software to generate the lollipop diagram and to calculate the efficiency of bisulfite conversion. Analysis of non-CpG cytosines indicated the efficiency of bisulfite conversion at ∼99%. The lollipop diagram presented the percentage of methylation of each CpG. The overall methylation percentage indicates the total proportion of methylated CpGs in this region taking into account all sequenced alleles.

Cancer is characterized by "methylation imbalance," in which genome-wide hypomethylation is accompanied by localized hypermethylation, and an increase in the expression of DNA methyltransferase [43]. DNA methylation is one of several cellular epigenetic mechanisms that control gene expression. To evaluate the contribution of epigenetic silencing to the reduced *CHD5* gene expression observed in leukemia cells, we investigated whether the identified *CHD5* promoter regulatory element was methylated in leukemia cell lines. The methylation status in K-562, KG-1a, HL-60 and Jurkat cells was examined by BGS of the 39 CpG dinucleotides located in the –560 to –240 region of the *CHD5* promoter (Figure 3). NMCs were included in this experiment for comparison. All CpG dinucleotides were almost methylated completely in these cells (methylation density in K-562, KG-1a, HL-60 and Jurkat cells was 99.2%, 99.4%, 97.4% and 76.9%, respectively). In contrast, the methylation density in NMCs was much lower (6.99%) (Figure 4).

### Activation of CHD5 gene expression by epigenetic modulatory drugs in leukemia cell lines

**Figure 5:**
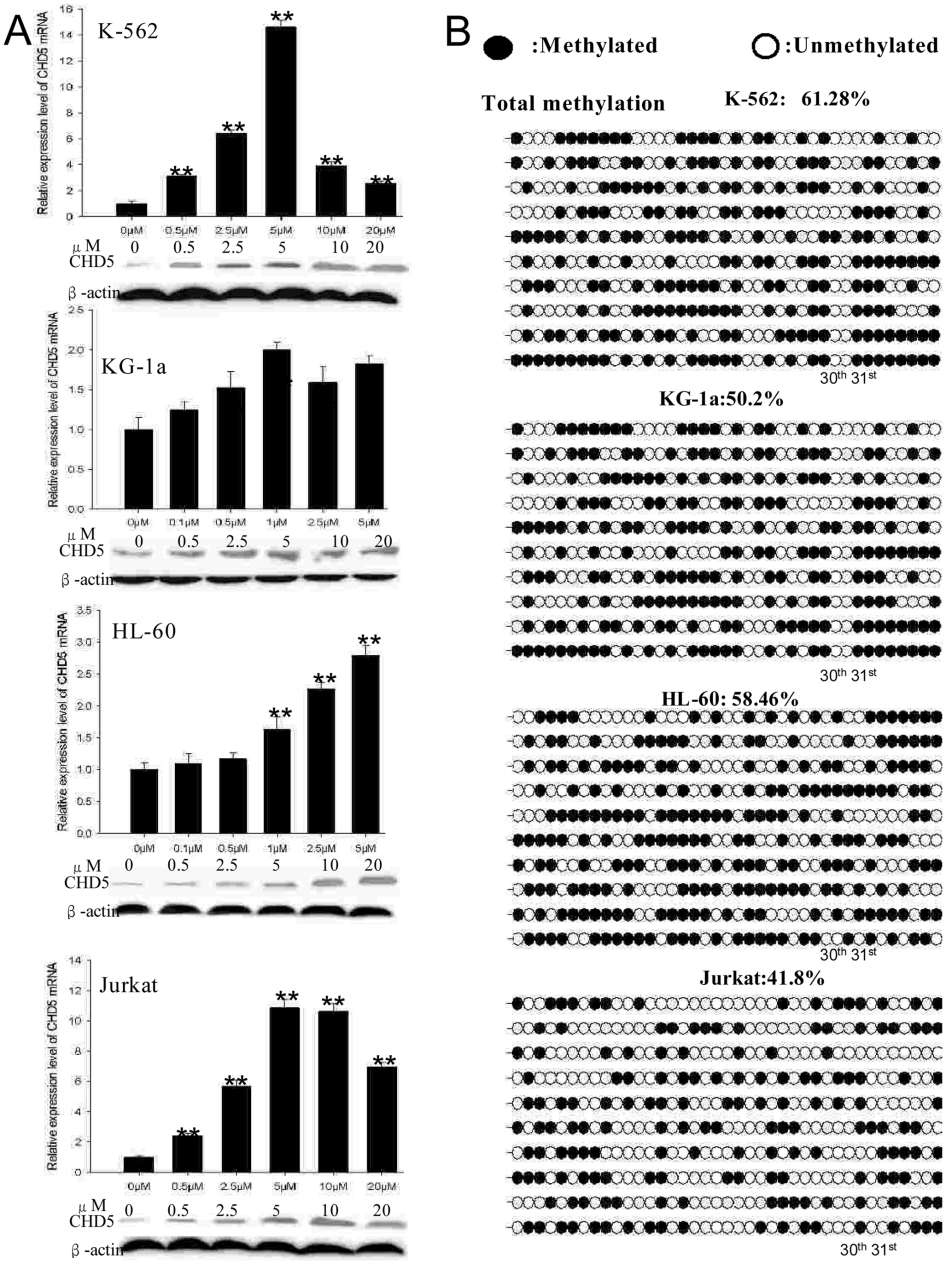
The methylation status of *CHD5* promoter and corresponding *CHD5* expression in leukemia cell lines treated with DAC. K-562, Jurkat, HL-60 and KG-1a cells were treated with DNA methyltransferase inhibitor DAC at the indicated concentrations. *CHD5* expression (A) was analyzed by qPCR (top) and western blotting (bottom). Data are shown as mean ± SD. The methylation status (B) of the –560 to –240 region of the *CHD5* promoter was analyzed by BGS.

The association between these epigenetic aberrations and the putative transcriptional inactivation of CHD5 gene was assessed in leukemia cell lines. Cells were grown in increasing concentrations of DAC and CHD5 expression was measured using qRT-PCR and western blotting. DAC elicited a dose-dependent induction of CHD5 expression in all cell lines (Figure 5 A; P<0.01) that was most dramatic in K-562 and Jurkat cells (one order of magnitude higher expression), cells that initially exhibited the lowest initial *CHD5* expression. To confirm the demethylation effect of DAC, BGS was performed on treated cell lines. Overall methylation of the *CHD5* promoter in was reduced in K-562 (99.2% to 61.28%), KG-1a (99.4% to 50.02%), HL-60 (97.4% to 58.46%) and Jurkat cells (76.9% to 41.8%) treated with DAC (Figure 5 B). Furthermore, methylation density of the 30^th^ and 31^st^ CpG sites (Figure S2) was significantly decreased in DAC-treated K-562 and Jurkat cells, which exhibited the most significant restoration of *CHD5* transcript levels (Figure 5 B).

### Methylation status of *CHD5* promoter in leukemia samples

**Figure 6:**
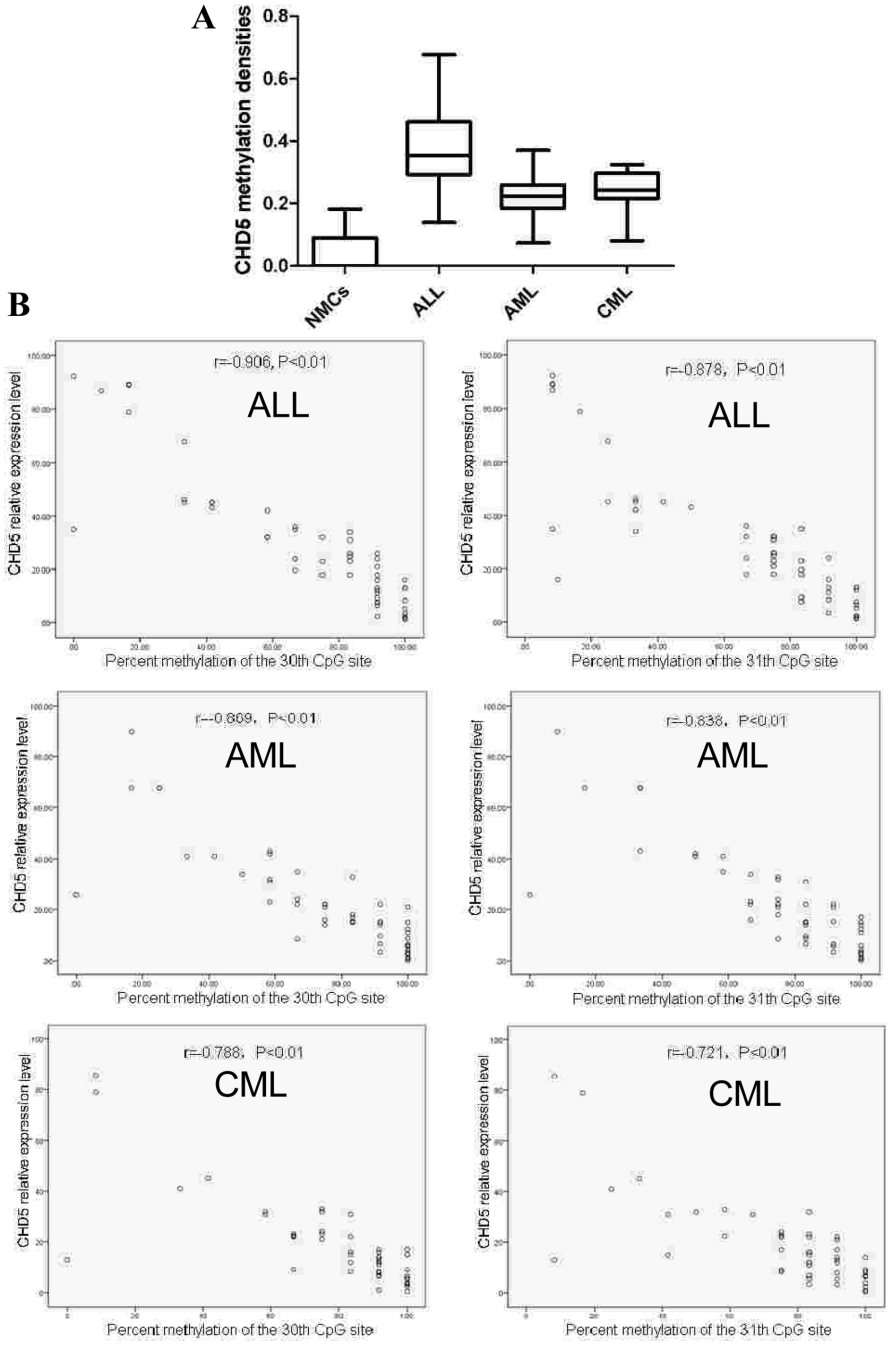
Relationship between *CHD5* expression level and *CHD5* promoter methylation status. The CpG island located at –560 to –240 was analyzed by BGS. The distribution of overall methylation percentage of *CHD5* promoter in ALL, AML and CML and NMCs was calculated (A). The correlation between the methylation level of 30^th^ and 31^st^ CpG sites and *CHD5* expression level was analyzed by Spearman’s correlation (B).

To determine whether the identified *CHD5* promoter regulatory element was also hypermethylated in leukemia samples exhibiting reduced *CHD5* expression, the methylation of the CpG island (–560 to –240) was methylated. Median methylation densities in ALL, AML, CML and NMCs were 38.5% (range, 0.14–0.677), 22.9% (range, 0.167–0.296), 24.5% (range, 0.189–0.297) and 7.2% (range, 0–0.182), respectively (Figure 6 A; P<0.01). Furthermore, the degree of methylation at the 30^th^ and 31^st^ CpG sites were inversely correlated with the *CHD5* expression level (Figure 6 B; P<0.01).

### Repression of human *CHD5* promoter activity by DNA methylation

**Figure 7:**
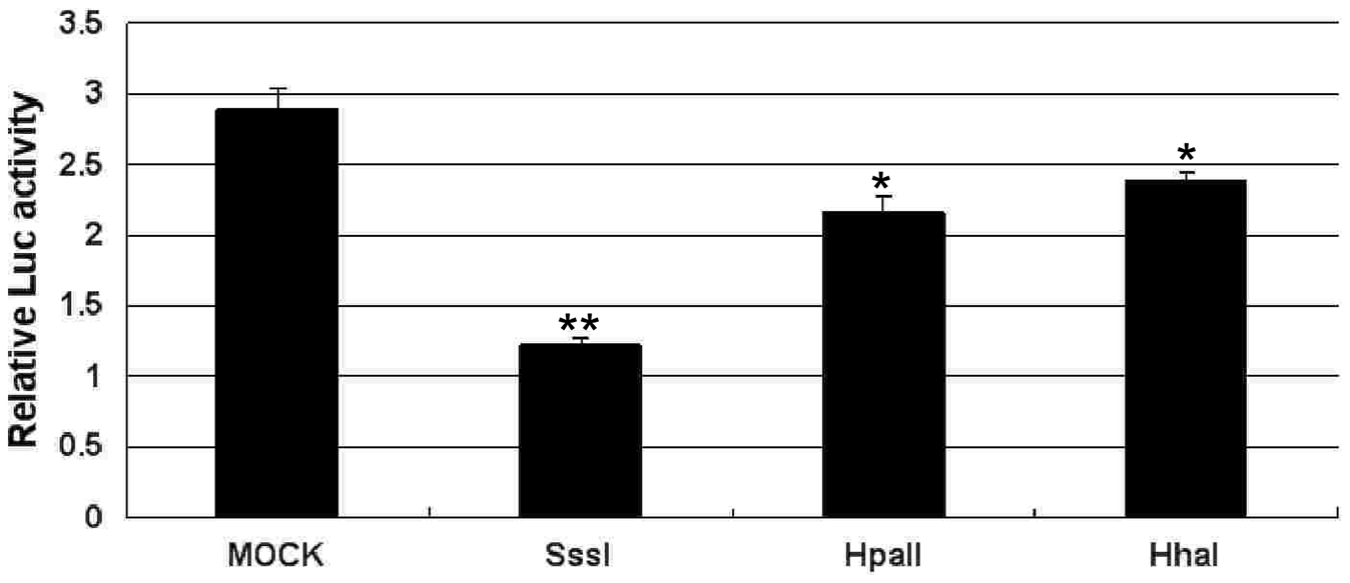
Repression of *CHD5* promoter activity by DNA methylation. A set of reporter constructs of methylated or mock-methylated fragments of the –560 to –240 region were measured for luciferase activity in K-562 cells. The relative Luc activities of methylated and mock *CHD5* promoters were normalized to Renilla luciferase activity. All data are presented as mean ± SD. P<0.001 was considered statistically significant (**).

To examine whether DNA methylation directly represses *CHD5* promoter activity, we cloned the identified *CHD5* promoter regulatory element (–560 to –240) into a luciferase reporter construct. We methylated the cloned insert using *Sss*I methylase (methylation of 39 CpGs), *Hpa*II methyltransferase (methylation of 6 CpGs) or *Hha*I methylase (methylation of 8 CpGs). Furthermore, the 30^th^ and 31^st^ CpG sites were methylated by *Sss*I methylase and *Hpa*II methyltransferase, but not by *Hha*I methylase. Proper methylation of inserts was confirmed by digestion with the restriction enzymes *Mcr*BC, *Hpa*II and *Hha*I. *CHD5* promoter activity was tested by transfection of the luciferase construct containing methylated *CHD5* promoter. Methylation with *Sss*I, *Hpa*II or *Hha*I methylases repressed *CHD5* promoter activity in K-562. Repression was methylation dose dependent in that *Sss*I, which methylates all CpG sites, exhibited the greatest repression, *Hpa*II, which methylates 6 CpG sites, exhibited moderate repression, and *Hha*I, which methylates 6 or 8 CpG sites, exhibited the least repression of *CHD5* promoter activity (Figure 7; P<0.01).

### Identification of the AP2 binding site in the *CHD5* promoter regulatory element

After identifying an important regulatory element at –500 to –200 of the *CHD5* promoter and confirming that methylation of this site regulates expression, we examined the promoter sequence for known TBSs using WWW Promoter Scan [40] and TFSEARCH software [41]. The analysis identified a putative AP2 binding site at –369 to –357, a region that includes the 30^th^ and 31^st^ CpG sites. It is known that AP2 binding is methylation sensitive and that methylation of this site regulates transcription [44], [45], [46].

**Figure 8:**
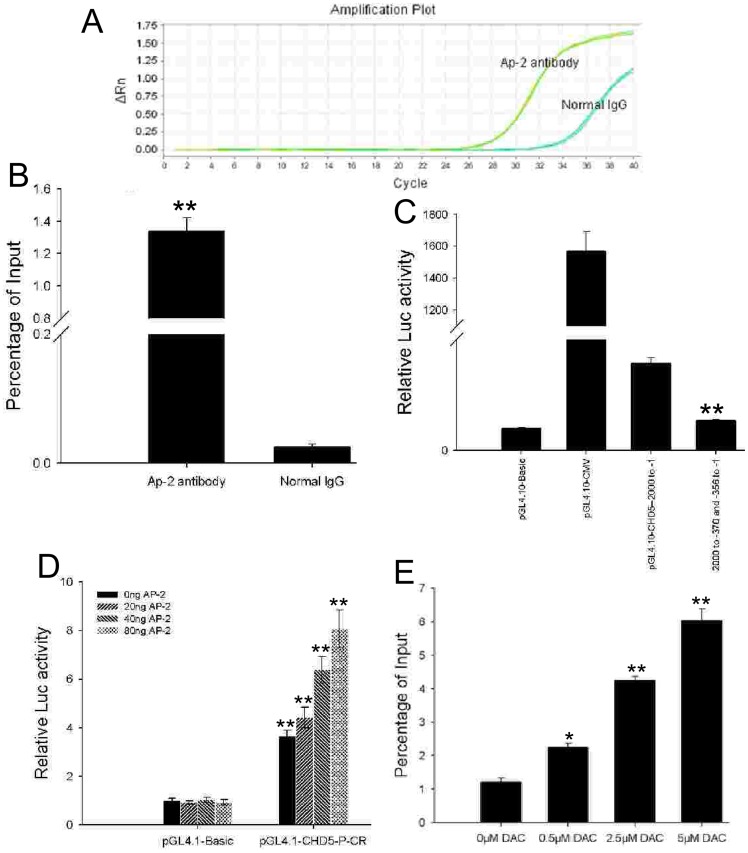
Identification of the AP2 binding site in the CHD5 promoter regulatory element. ChIP-qPCR assay indicated that AP2 binds to the identified regulatory element of the *CHD5* promoter (A, B). Normal IgG was used as a negative control. The pGL4.10-CHD5-2000 to -370 and pGL4.10-CHD5-356 vectors, in which the AP2 binding site was deleted, were measured for luciferase activity in K-562 cells (C). The pGL4.1-CHD5-P-CR vector, harboring the –560 to –240 region, was measured for luciferase activity in K-562 cells (D). ChIP-qPCR analysis showed that DAC treatment enhanced AP2 binding to the *CHD5* promoter. All data are presented as mean ± SD. P<0.001 was considered statistically significant (**).

The AP2 binding site was confirmed by ChIP-qPCR using an AP2 antibody and luciferase constructs with *CHD5* promoter sequences mutated at the AP2 site (Figure 8A B C; P<0.01). *In vitro* transcription-factor activity studies indicated that *CHD5* promoter activity gradually increased following AP2 up-regulation (Figure 8D; P<0.01). To examine whether epigenetic change affects the binding of the AP2 to the *CHD5* promoter region, ChIP-qPCR analysis using an AP2-specific antibody was performed. AP2 binding increased significantly in K-562 cells upon treatment with DAC (Figure 8E; P<0.01). We conclude that the increase in AP2 binding to the *CHD5* promoter did not result from AP2 up-regulation, as DAC treatment did not significantly increase AP2 expression levels (Figure S3). These data provide functional evidence that decreased AP2 binding to the *CHD5* promoter in K-562 cells resulting from hypermethylation contributes to the suppression of *CHD5* expression. Specifically, these results support a link between dense CpG island hypermethylation in the *CHD5* promoter and transcriptional inactivation of the gene.

## Discussion

Several cancer cell lines and tumors from neural (neuroblastoma and glioma) [47], [48], [49] and epithelial (colon and breast) [12], [14], [47] lineages have 1p36 deletions that encompass the *CHD5* gene. The 1p36 region is also deleted in 5% of all hematopoietic malignancies, including AML [8], CML [9] and non-Hodgkin’s lymphoma [10]. Despite the fact that 1p36 deletions in other hematopoietic malignancies do not include the *CHD5* gene [8], [9], [10], *CHD5* deficiency has been shown to be important in these cases [35], [39]. Recent studies have indicated that *CHD5* expression is epigenetically silenced by hypermethylation of the *CHD5* promoter in cancer cells in which the *CHD5* gene is not deleted [17], [18], [19], [20], [21].

In this study, we found that *CHD5* is down-regulated in human leukemia cell lines as well as clinical samples. This observation suggests that *CHD5* could be used as a candidate gene for developing a biomarker panel for hematopoietic malignancies.

It has been reported that the *CHD5* promoter was strongly methylated in the –780 to –450 region (Figure S2) in neuroblastoma cell lines with 1p36 deletions, but not cell lines with two copies of the *CHD5* gene [36]. The methylated region (Figure S2) we identified (–500 to –200) is proximal to the region described in this report. It remains to be seen whether tissue-specific differences exist in the methylation of this region and modulate transcriptional regulation of the *CHD5* gene.

Several results support the hypothesis that the methylation of the *CHD5* promoter is responsible for the reduced expression observed in leukemia cells. *CHD5* expression was up-regulated when cells were grown in the presence of DNA methyltransferase inhibitor DAC. *In vitro* methylation of the *CHD5* promoter directly repressed promoter activity. We further showed that decreased expression of CHD5 in malignant tissues was significantly associated with hypermethylation of the CHD5 promoter, showing a relationship between methylation and reduced CHD5 expression in leukemia. Furthermore, the methylation level of 30^th^ and 31^st^ CpG sites, both located in the AP2 binding site, correlated inversely with *CHD5* expression. AP2 binding site is known to be methylation sensitive and that methylation of the site leads to transcriptional repression. The role of methylation in AP2 binding of the *CHD5* promoter was investigated with a ChIP-qPCR assay, which identified AP2 as a candidate transcription factor involved in *CHD5* expression. In addition, our *in vitro* transcription factor activity studies revealed an important effect for AP2 on *CHD5* expression. Furthermore, it was shown that DAC treatment increases the recruitment of AP2 to the *CHD5* promoter.

The findings of our study suggest that *CHD5* promoter methylation is a mechanism for controlling *CHD5* expression in human cancer. However, we did find some discordance between methylation and expression of *CHD5* gene. For example, the KG-1a cell line had the most hypermethylated promoter but expressed more *CHD5* than the other cell lines. In addition, reduced *CHD5* expression was not accompanied with hypermethylation of the *CHD5* promoter in some leukemia samples. These observations suggest that there are multiple mechanisms involve in the regulation of *CHD5* expression. Although homozygous genetic inactivation of *CHD5* was not observed in neuroblastomas [36], the down-regulation of *CHD5* expression resulted from the deletion of a *CHD5* allele as well as from strong methylation of the *CHD5* promoter [36]. Mutation and promoter hypermethylation of the *CHD5* gene was observed in ovarian cancer exhibiting reduced *CHD5* expression [24]. The low expression of *CHD5* in colorectal cancer is correlated with hypermethylation of the CpG island in the *CHD5* promoter and translational repression mediated by *miR-211* [50]. Recently, it has been demonstrated that *CHD5* promoter hypermethylation results in low or absent CHD5 protein levels in lung cancer [21] and that elevated *JMJD2A* expression observed in lung tumors of both human and mouse could contribute to the reduction of *CHD5* levels [31].

*CHD5* promoter hypermethylation may be associated with heterozygous loss of *CHD5*, mutation of the *CHD5* gene and transcriptional repression mediated by other factors in our leukemia samples and cell lines. For example, *CHD5* promoter hypermethylation and heterozygous loss of *CHD5* was observed in HL-60 and K-562 cells (Figure S4). In fact, the mechanism of *CHD5* expression regulation in different tissue types and developmental stages may be far more complicated than anticipated. Nonetheless, this epigenetic mechanism could reduce expression enough to silence *CHD5* gene functionally.

In summary, we have shown that *CHD5* is down-regulated in leukemia cells by promoter methylation, which suggests that *CHD5* is a potential prognostic biomarker that may enrich therapeutic options. Additionally, we identified the AP2 transcription factor as a new powerful regulator for *CHD5* expression. The data obtained from this study contribute to our understanding of the mechanisms regulating CHD5 gene expression in hematopoietic tumorigenesis, and facilitate optimization of anti-tumor therapy focused on repression of *CHD5* expression.

## Supporting Information

Figure S1 **Putative** ***CHD5*** **promoter.** Analysis of the human *CHD5* gene using UCSC Genome Browser software indicated that the putative *CHD5* promoter was probably located 2000 bp upstream of TSS and overlapped with a CpG island.
(TIF)

Figure S2 **The graphical representation of the methylated region.** The methylated region that Zhao *et al* identified at −560 to −240 is somewhat proximal to the region that Fujita et al have identified at −780 to −450. The DNA sequence of −780 to −450 marked by underline was partially overlapped with DNA sequence of −560 to −240 marked by box. These CpG sites is marked by green shading. The sequence of all CpG sites located at −560 to −240 marked by sequential number.
(TIF)

Figure S3 **AP2 expression in K-56 cell lines treated with DAC.** K-562 cells were treated with DNA methyltransferase inhibitor DAC at the indicated concentrations. AP2 expression was determined by qRT-PCR (A) and western blotting (B). β-Actin was detected as an internal control. All data are presented as mean ± SD.
(TIF)

Figure S4 **Visualization of chromosomal aberrations of K-562 and HL-60 cell lines.** The SKY/M-FISH for K-562 (A) and HL-60 (B) cell lines were quoted from SKY/M-FISH and CGH Database at NCBI (NCI and NCBI SKY/M-FISH and CGH Database (2001), http://www.ncbi.nlm.nih.gov/sky/skyweb.cgi).
(TIF)

Table S1 **Patient samples.**
(DOC)
