# Supplementary figures and images for: Silencing of CHD5 Gene by Promoter Methylation in Leukemia
Source: PLoS One. 2014 Jan 13;9(1):e85172. doi: 10.1371/journal.pone.0085172 (PMC3890315; doi:10.1371/journal.pone.0085172)

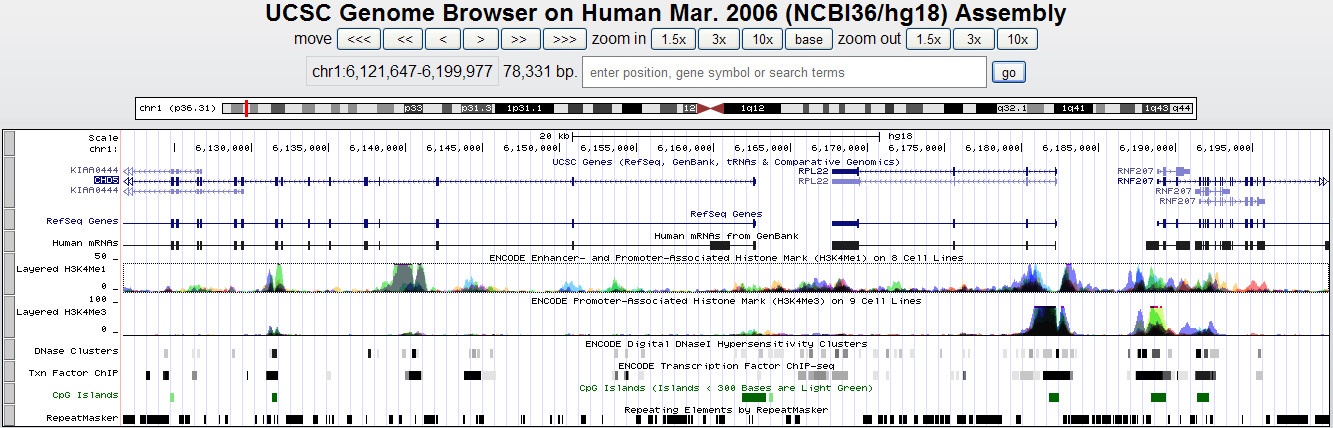

Supplement: Figure S1 — Putative CHD5 promoter. Analysis of the human CHD5 gene using UCSC Genome Browser software indicated that the putative CHD5 promoter was probably located 2000 bp upstream of TSS and overlapped with a CpG island. (TIF) [file pone.0085172.s001.tif]

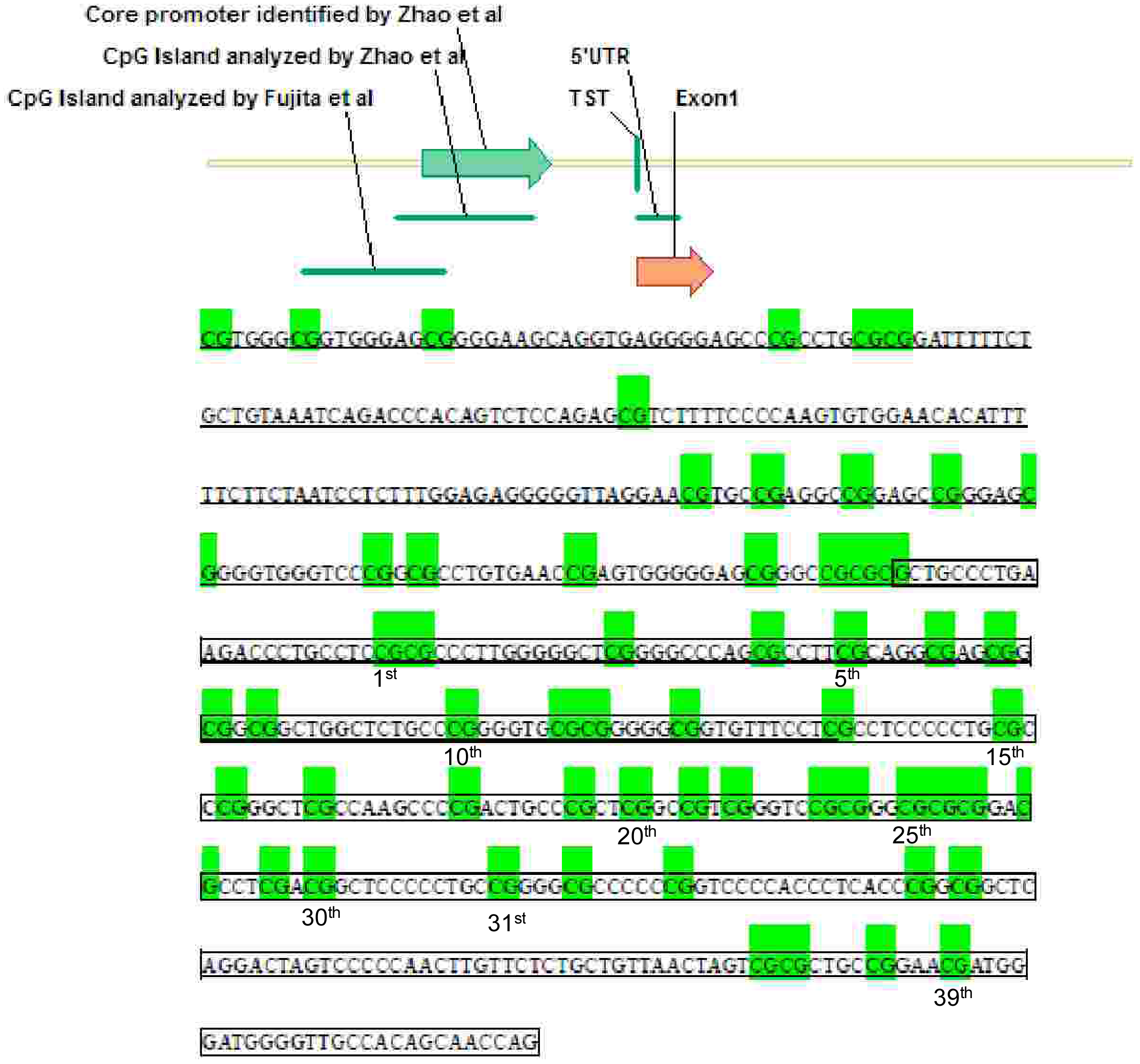

Supplement: Figure S2 — The graphical representation of the methylated region. The methylated region that Zhao et al identified at −560 to −240 is somewhat proximal to the region that Fujita et al have identified at −780 to −450. The DNA sequence of −780 to −450 marked by underline was partially overlapped with DNA sequence of −560 to −240 marked by box. These CpG sites is marked by green shading. The sequence of all CpG sites located at −560 to −240 marked by sequential number. (TIF) [file pone.0085172.s002.tif]

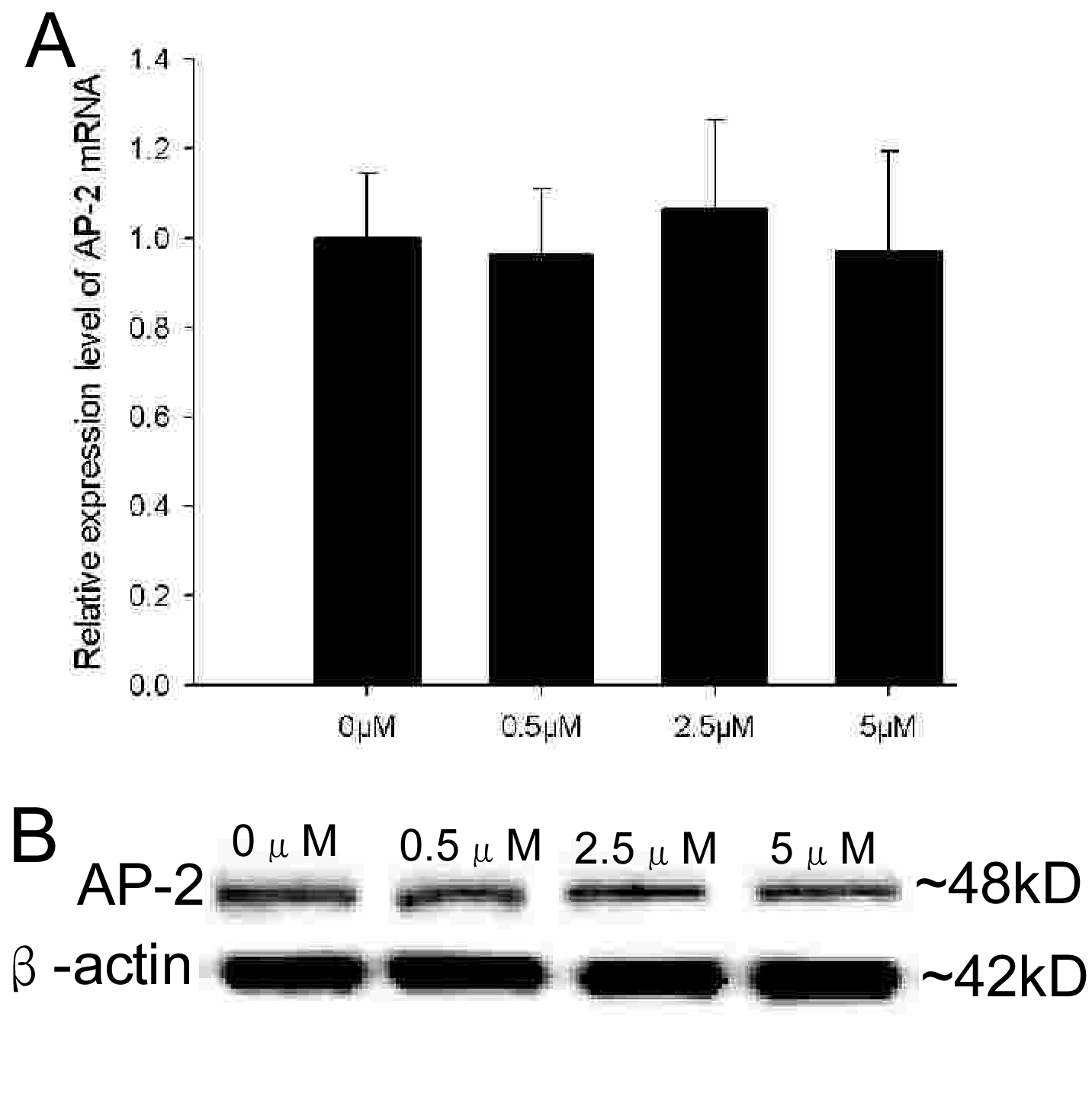

Supplement: Figure S3 — AP2 expression in K-56 cell lines treated with DAC. K-562 cells were treated with DNA methyltransferase inhibitor DAC at the indicated concentrations. AP2 expression was determined by qRT-PCR (A) and western blotting (B). β-Actin was detected as an internal control. All data are presented as mean ± SD. (TIF) [file pone.0085172.s003.tif]

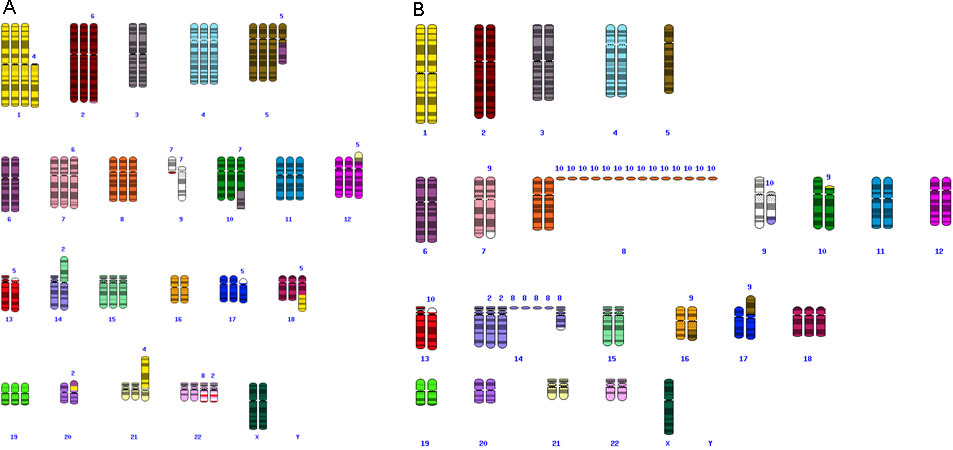

Supplement: Figure S4 — Visualization of chromosomal aberrations of K-562 and HL-60 cell lines. The SKY/M-FISH for K-562 (A) and HL-60 (B) cell lines were quoted from SKY/M-FISH and CGH Database at NCBI (NCI and NCBI SKY/M-FISH and CGH Database (2001), http://www.ncbi.nlm.nih.gov/sky/skyweb.cgi). (TIF) [file pone.0085172.s004.tif]
