# Supplementary material for: Silencing of CHD5 Gene by Promoter Methylation in Leukemia
Source: PLoS One. 2014 Jan 13;9(1):e85172. doi: 10.1371/journal.pone.0085172 (PMC3890315; doi:10.1371/journal.pone.0085172)
Supplement: Table S1 — Patient samples. (DOC) [file pone.0085172.s005.doc]

**Table S1. Patient samples**

| Sample | Age | sex | Staging | % of  Ph positive  cells | CHD5 level | The methylation rate of 30th CpG island | The methylation rate of 31th CpG island |
| --- | --- | --- | --- | --- | --- | --- | --- |
| ALL-1 | 41 | M | primary | 99% | 18 | 83.33333333 | 66.66666667 |
| ALL-2 | 25 | F | primary | 93% | 19.66666667 | 66.66666667 | 83.33333333 |
| ALL-3 | 56 | M | primary | 94% | 8.333333333 | 100 | 91.66666667 |
| ALL-4 | 78 | F | primary | 98% | 35 | 0 | 8.333333333 |
| ALL-5 | 16 | F | primary | 97% | 16 | 91.66666667 | 91.66666667 |
| ALL-6 | 26 | F | primary | 95% | 18 | 75 | 83.33333333 |
| ALL-7 | 23 | F | primary | 93% | 7.666666667 | 91.66666667 | 83.33333333 |
| ALL-8 | 35 | F | primary | 95% | 23 | 75 | 75 |
| ALL-9 | 43 | M | primary | 99% | 89.2 | 16.66666667 | 8.333333333 |
| ALL-10 | 36 | F | primary | 95% | 43 | 41.66666667 | 50 |
| ALL-11 | 48 | F | primary | 93% | 6.56 | 91.66666667 | 100 |
| ALL-12 | 25 | M | primary | 92% | 24 | 66.66666667 | 66.66666667 |
| ALL-13 | 52 | M | primary | 94% | 18 | 91.66666667 | 75 |
| ALL-14 | 26 | M | primary | 94% | 32 | 75 | 75 |
| ALL-15 | 46 | F | primary | 96% | 9.34 | 91.66666667 | 83.33333333 |
| ALL-16 | 55 | F | primary | 93% | 5.34 | 100 | 100 |
| ALL-17 | 53 | F | primary | 92% | 13 | 91.66666667 | 100 |
| ALL-18 | 56 | F | primary | 92% | 92.3 | 0 | 8.333333333 |
| ALL-19 | 58 | F | primary | 93% | 26 | 83.33333333 | 75 |
| ALL-20 | 45 | M | primary | 90% | 35 | 66.66666667 | 83.33333333 |
| ALL-21 | 34 | F | primary | 89% | 7.68 | 91.66666667 | 100 |
| ALL-22 | 36 | M | primary | 92% | 42 | 58.33333333 | 33.33333333 |
| ALL-23 | 47 | F | primary | 92% | 45 | 41.66666667 | 41.66666667 |
| ALL-24 | 46 | F | primary | 89% | 32 | 58.33333333 | 75 |
| ALL-25 | 38 | F | primary | 92% | 67.8 | 33.33333333 | 25 |
| ALL-26 | 39 | M | primary | 93% | 21 | 91.66666667 | 75 |
| ALL-27 | 52 | F | primary | 92% | 16 | 100 | 10 |
| ALL-28 | 19 | F | primary | 88% | 2.34 | 91.66666667 | 100 |
| ALL-29 | 46 | M | primary | 97% | 12 | 91.66666667 | 100 |
| ALL-30 | 57 | F | primary | 95% | 25 | 83.33333333 | 75 |
| ALL-31 | 52 | M | primary | 95% | 88.9 | 16.66666667 | 8.333333333 |
| ALL-32 | 55 | M | primary | 91% | 36 | 66.66666667 | 66.66666667 |
| ALL-33 | 53 | F | primary | 92% | 45 | 33.33333333 | 33.33333333 |
| ALL-34 | 51 | F | primary | 92% | 13 | 100 | 91.66666667 |
| ALL-35 | 46 | F | primary | 94% | 23 | 83.33333333 | 83.33333333 |
| ALL-36 | 44 | F | primary | 92% | 78.9 | 16.66666667 | 16.66666667 |
| ALL-37 | 38 | M | primary | 95% | 34 | 83.33333333 | 33.33333333 |
| ALL-38 | 39 | F | primary | 95% | 3.56 | 100 | 91.66666667 |
| ALL-39 | 33 | F | primary | 96% | 45 | 41.66666667 | 25 |
| ALL-40 | 36 | M | primary | 91% | 46 | 33.33333333 | 33.33333333 |
| ALL-41 | 29 | M | primary | 96% | 32 | 58.33333333 | 66.66666667 |
| ALL-42 | 41 | M | primary | 95% | 24 | 91.66666667 | 91.66666667 |
| ALL-43 | 43 | M | primary | 94% | 11 | 91.66666667 | 91.66666667 |
| ALL-44 | 38 | F | primary | 93% | 2.35 | 100 | 100 |
| ALL-45 | 25 | F | primary | 95% | 26 | 91.66666667 | 75 |
| ALL-46 | 23 | M | primary | 92% | 1.67 | 100 | 100 |
| ALL-47 | 44 | F | primary | 91% | 31 | 83.33333333 | 75 |
| ALL-48 | 46 | M | primary | 89% | 13 | 100 | 100 |
| ALL-49 | 37 | F | primary | 87% | 1.23 | 100 | 100 |
| ALL-50 | 32 | F | primary | 86% | 86.8 | 8.333333333 | 8.333333333 |
| AML-1 | 23 | M | primary | 91% | 12.33333333 | 100 | 100 |
| AML-2 | 25 | M | primary | 92% | 15.33333333 | 83.33333333 | 91.66666667 |
| AML-3 | 63 | M | primary | 93% | 25.66666667 | 0 | 0 |
| AML-4 | 64 | F | primary | 94% | 18 | 83.33333333 | 75 |
| AML-5 | 25 | F | primary | 95% | 17 | 83.33333333 | 100 |
| AML-6 | 46 | F | primary | 96% | 15 | 100 | 83.33333333 |
| AML-7 | 35 | F | primary | 91% | 6 | 100 | 100 |
| AML-8 | 36 | F | primary | 93% | 8.666666667 | 66.66666667 | 75 |
| AML-9 | 37 | F | primary | 92% | 23 | 58.33333333 | 66.66666667 |
| AML-10 | 46 | M | primary | 94% | 34 | 50 | 66.66666667 |
| AML-11 | 45 | M | primary | 89% | 6.52 | 100 | 91.66666667 |
| AML-12 | 54 | M | primary | 87% | 21 | 75 | 75 |
| AML-13 | 55 | M | primary | 92% | 15 | 91.66666667 | 83.33333333 |
| AML-14 | 61 | M | primary | 93% | 5.67 | 100 | 91.66666667 |
| AML-15 | 13 | M | primary | 98% | 43 | 58.33333333 | 33.33333333 |
| AML-16 | 18 | M | primary | 99% | 89.65 | 16.66666667 | 8.333333333 |
| AML-17 | 16 | F | primary | 97% | 16 | 75 | 66.66666667 |
| AML-18 | 21 | F | primary | 96% | 3.45 | 100 | 91.66666667 |
| AML-19 | 39 | F | primary | 95% | 24 | 66.66666667 | 75 |
| AML-20 | 41 | F | primary | 95% | 2.87 | 100 | 100 |
| AML-21 | 45 | M | primary | 96% | 35 | 66.66666667 | 58.33333333 |
| AML-22 | 62 | M | primary | 96% | 67.8 | 25 | 33.33333333 |
| AML-23 | 23 | M | primary | 94% | 9.65 | 91.66666667 | 83.33333333 |
| AML-24 | 14 | M | primary | 94% | 41 | 41.66666667 | 50 |
| AML-25 | 23 | F | primary | 95% | 0.34 | 100 | 100 |
| AML-26 | 56 | M | primary | 95% | 42 | 58.33333333 | 50 |
| AML-27 | 55 | F | primary | 97% | 1.23 | 100 | 100 |
| AML-28 | 51 | M | primary | 93% | 22 | 66.66666667 | 75 |
| AML-29 | 52 | F | primary | 92% | 6.7 | 91.66666667 | 83.33333333 |
| AML-30 | 45 | M | primary | 91% | 31 | 58.33333333 | 83.33333333 |
| AML-31 | 42 | F | primary | 90% | 22 | 75 | 91.66666667 |
| AML-32 | 41 | M | primary | 90% | 3.45 | 91.66666667 | 100 |
| AML-33 | 31 | F | primary | 98% | 11 | 100 | 100 |
| AML-34 | 32 | F | primary | 94% | 8.76 | 100 | 83.33333333 |
| AML-35 | 33 | M | primary | 96% | 14 | 91.66666667 | 100 |
| AML-36 | 25 | M | primary | 91% | 2.65 | 100 | 100 |
| AML-37 | 26 | M | primary | 97% | 22 | 75 | 66.66666667 |
| AML-38 | 37 | M | primary | 94% | 67.89 | 25 | 16.66666667 |
| AML-39 | 26 | M | primary | 97% | 15 | 83.33333333 | 83.33333333 |
| AML-40 | 32 | F | primary | 94% | 41 | 33.33333333 | 58.33333333 |
| AML-41 | 18 | F | primary | 95% | 4.32 | 100 | 100 |
| AML-42 | 29 | F | primary | 91% | 32 | 58.33333333 | 75 |
| AML-43 | 32 | M | primary | 93% | 15 | 83.33333333 | 100 |
| AML-44 | 33 | F | primary | 92% | 33 | 83.33333333 | 75 |
| AML-45 | 45 | M | primary | 91% | 67.89 | 16.66666667 | 33.33333333 |
| AML-46 | 46 | F | primary | 91% | 14 | 75 | 83.33333333 |
| AML-47 | 47 | M | primary | 92% | 15 | 91.66666667 | 83.33333333 |
| AML-48 | 49 | F | primary | 91% | 0.89 | 100 | 100 |
| AML-49 | 26 | M | primary | 90% | 21 | 100 | 91.66666667 |
| AML-50 | 25 | F | primary | 89% | 22 | 91.66666667 | 83.33333333 |
| CML-1 | 64 | F | primary | 99% | 9 | 100 | 100 |
| CML-2 | 61 | F | primary | 98% | 16 | 83.33333333 | 83.33333333 |
| CML-3 | 26 | M | primary | 97% | 15 | 83.33333333 | 83.33333333 |
| CML-4 | 65 | M | primary | 88% | 12 | 83.33333333 | 83.33333333 |
| CML-5 | 48 | M | primary | 92% | 14 | 91.66666667 | 91.66666667 |
| CML-6 | 45 | M | primary | 93% | 13 | 0 | 8.333333333 |
| CML-7 | 46 | F | primary | 95% | 9 | 66.66666667 | 75 |
| CML-8 | 48 | F | primary | 94% | 22.33333333 | 66.66666667 | 58.33333333 |
| CML-9 | 32 | F | primary | 94% | 23 | 66.66666667 | 75 |
| CML-10 | 23 | F | primary | 95% | 8.2 | 91.66666667 | 100 |
| CML-11 | 26 | M | primary | 96% | 24 | 75 | 75 |
| CML-12 | 36 | M | primary | 92% | 2.4 | 100 | 100 |
| CML-13 | 39 | M | primary | 90% | 31 | 58.33333333 | 41.66666667 |
| CML-14 | 47 | F | primary | 92% | 78.9 | 8.333333333 | 16.66666667 |
| CML-15 | 32 | F | primary | 99% | 3.8 | 100 | 100 |
| CML-16 | 35 | M | primary | 97% | 22 | 66.66666667 | 75 |
| CML-17 | 33 | M | primary | 95% | 12 | 91.66666667 | 83.33333333 |
| CML-18 | 36 | F | primary | 94% | 6.7 | 91.66666667 | 83.33333333 |
| CML-19 | 30 | F | primary | 91% | 33 | 75 | 58.33333333 |
| CML-20 | 15 | M | primary | 92% | 8.3 | 83.33333333 | 75 |
| CML-21 | 62 | M | primary | 93% | 16 | 91.66666667 | 83.33333333 |
| CML-22 | 23 | F | primary | 91% | 21 | 75 | 91.66666667 |
| CML-23 | 46 | F | primary | 90% | 17 | 91.66666667 | 75 |
| CML-24 | 58 | M | primary | 91% | 3.4 | 100 | 83.33333333 |
| CML-25 | 26 | M | primary | 98% | 45 | 41.66666667 | 33.33333333 |
| CML-26 | 25 | F | primary | 92% | 32 | 58.33333333 | 50 |
| CML-27 | 34 | F | primary | 93% | 22 | 66.66666667 | 83.33333333 |
| CML-28 | 26 | F | primary | 92% | 0.98 | 91.66666667 | 100 |
| CML-29 | 28 | F | primary | 99% | 12 | 91.66666667 | 91.66666667 |
| CML-30 | 34 | M | primary | 94% | 11 | 91.66666667 | 83.33333333 |
| CML-31 | 35 | F | primary | 95% | 13 | 91.66666667 | 91.66666667 |
| CML-32 | 38 | M | primary | 93% | 6.7 | 91.66666667 | 100 |
| CML-33 | 62 | F | primary | 92% | 32 | 75 | 83.33333333 |
| CML-34 | 31 | F | primary | 99% | 22 | 83.33333333 | 75 |
| CML-35 | 25 | M | primary | 91% | 5.6 | 100 | 83.33333333 |
| CML-36 | 61 | M | primary | 91% | 14 | 91.66666667 | 100 |
| CML-37 | 26 | M | primary | 94% | 6.4 | 100 | 100 |
| CML-38 | 28 | M | primary | 99% | 23 | 75 | 83.33333333 |
| CML-39 | 34 | F | primary | 94% | 85.6 | 8.333333333 | 8.333333333 |
| CML-40 | 37 | F | primary | 97% | 7.8 | 91.66666667 | 91.66666667 |
| CML-41 | 46 | F | primary | 91% | 41 | 33.33333333 | 25 |
| CML-42 | 51 | F | primary | 92% | 3.2 | 100 | 91.66666667 |
| CML-43 | 36 | F | primary | 99% | 31 | 83.33333333 | 66.66666667 |
| CML-44 | 48 | M | primary | 98% | 0.45 | 100 | 100 |
| CML-45 | 51 | M | primary | 97% | 22 | 66.66666667 | 91.66666667 |
| CML-46 | 62 | F | primary | 89% | 15 | 100 | 41.66666667 |
| CML-47 | 25 | M | primary | 97% | 6.6 | 91.66666667 | 100 |
| CML-48 | 23 | F | primary | 94% | 17 | 100 | 91.66666667 |
| CML-49 | 18 | F | primary | 95% | 5.4 | 100 | 91.66666667 |
| CML-50 | 29 | M | primary | 93% | 15 | 91.66666667 | 83.33333333 |
